# eQTLs Regulating Transcript Variations Associated with Rapid Internode Elongation in Deepwater Rice

**DOI:** 10.3389/fpls.2017.01753

**Published:** 2017-10-13

**Authors:** Takeshi Kuroha, Keisuke Nagai, Yusuke Kurokawa, Yoshiaki Nagamura, Miyako Kusano, Hideshi Yasui, Motoyuki Ashikari, Atsushi Fukushima

**Affiliations:** ^1^Bioscience and Biotechnology Center, Nagoya University, Nagoya, Japan; ^2^Department of Developmental Biology and Neurosciences, Graduate School of Life Sciences, Tohoku University, Sendai, Japan; ^3^Genome Resource Unit, National Institute of Agrobiological Sciences, Tsukuba, Japan; ^4^RIKEN Center for Sustainable Resource Science, Yokohama, Japan; ^5^Graduate School of Life and Environmental Sciences, University of Tsukuba, Tsukuba, Japan; ^6^Faculty of Agriculture, Kyushu University, Fukuoka, Japan

**Keywords:** quantitative trait locus, expression QTL, eQTL hotspots, ethylene response factor, submergence, abiotic stress, *Oryza sativa*

## Abstract

To avoid low oxygen, oxygen deficiency or oxygen deprivation, deepwater rice cultivated in flood planes can develop elongated internodes in response to submergence. Knowledge of the gene regulatory networks underlying rapid internode elongation is important for an understanding of the evolution and adaptation of major crops in response to flooding. To elucidate the genetic and molecular basis controlling their deepwater response we used microarrays and performed expression quantitative trait loci (eQTL) and phenotypic QTL (phQTL) analyses of internode samples of 85 recombinant inbred line (RIL) populations of non-deepwater (Taichung 65)- and deepwater rice (Bhadua). After evaluating the phenotypic response of the RILs exposed to submergence, confirming the genotypes of the populations, and generating 188 genetic markers, we identified 10,047 significant eQTLs comprised of 2,902 *cis*-eQTLs and 7,145 *trans*-eQTLs and three significant eQTL hotspots on chromosomes 1, 4, and 12 that affect the expression of many genes. The hotspots on chromosomes 1 and 4 located at different position from phQTLs detected in this study and other previous studies. We then regarded the eQTL hotspots as key regulatory points to infer causal regulatory networks of deepwater response including rapid internode elongation. Our results suggest that the downstream regulation of the eQTL hotspots on chromosomes 1 and 4 is independent, and that the target genes are partially regulated by *SNORKEL1* and *SNORKEL2* genes (*SK1*/*2*), key ethylene response factors. Subsequent bioinformatic analyses, including gene ontology-based annotation and functional enrichment analysis and promoter enrichment analysis, contribute to enhance our understanding of SK1/2-dependent and independent pathways. One remarkable observation is that the functional categories related to photosynthesis and light signaling are significantly over-represented in the candidate target genes of SK1/2. The combined results of these investigations together with genetical genomics approaches using structured populations with a deepwater response are also discussed in the context of current molecular models concerning the rapid internode elongation in deepwater rice. This study provides new insights into the underlying genetic architecture of gene expression regulating the response to flooding in deepwater rice and will be an important community resource for analyses on the genetic basis of deepwater responses.

## Introduction

Flooding, an environmental stress, affects the growth and development of most plants by limiting the exchange of gases such as oxygen and carbonic dioxide and reducing the light intensity; this reduces yield around the world. Some plants such as *Oryza, Rumex, Rorippa*, and *Echinochloa* genera can survive under this stressful condition by implementing specific strategies (Bailey-Serres and Voesenek, 2008). Flood-tolerant *Oryza sativa* (rice) developed two different strategies for surviving full and partial submergence (Voesenek and Bailey-Serres, 2015; Loreti et al., 2016). The ‘quiescent strategy’ allows flash-flood-tolerant rice to resume growth after the floodwater recedes (Bailey-Serres and Voesenek, 2010; Mickelbart et al., 2015). By the ‘escape strategy,’ the internodes of rice plants tolerant to deepwater flooding rapidly elongate as the water level rises (Nagai et al., 2010; Hattori et al., 2011). Consequently, deepwater rice can be grown in many flood-prone areas in South and Southeast Asia such as Bangladesh, Cambodia, and Thailand (Kende et al., 1998).

Many agronomically important traits, including tolerance to flash- and deepwater floods, tend to be governed by genes known as quantitative trait loci (QTL) (Quarrie et al., 1997; Price et al., 2002; Ashikari and Matsuoka, 2006; Salvi and Tuberosa, 2015). An Indian rice variety, FR13A that applies the quiescent strategy for submergence tolerance, harbors the major QTL *Submergence 1* (*SUB1*) (Nandi et al., 1997; Siangliw et al., 2003). *SUB1A*, a member of the APETALA2 (AP2)/Ethylene Response Factor (ERF) superfamily, has been cloned as the gene responsible for *SUB1* QTL (Xu et al., 2006). QTL analysis has also shown that the internode elongation trait in deepwater rice is regulated by three major QTLs (Tang et al., 2005; Hattori et al., 2007, 2008; Kawano et al., 2008). Subsequent work demonstrated that the QTL in chromosome 12, the major QTL controlling the deepwater response, harbors two transcription factors, *SNORKEL1* and *SNORKEL2* (*SK1*/*2*) (Hattori et al., 2009), members of the AP2/ERF superfamily. These findings suggest that deepwater rice acquired key regulators to adapt to different types of flood. However, our understanding of the gene regulatory networks underlying these traits in flood-tolerant rice in response to submergence remains far from complete and no other key regulators for submergence tolerance in deepwater rice have been identified.

In flash flood-tolerant rice, *Rumex, Rorippa*, and *Echinochloa*, large-scale transcriptome analysis using microarrays or RNA sequencing (RNA-seq) has been performed focusing on mechanism of flooding tolerance (Jung et al., 2010; Sasidharan et al., 2013; Van Veen et al., 2013; Nah et al., 2015). Progress in the acquisition of large-scale transcriptome data has greatly facilitated expression QTL (eQTL) analysis (Jansen and Nap, 2001; Kliebenstein, 2009; Druka et al., 2010; Cubillos et al., 2012a; Chitwood and Sinha, 2013); the gene expression level is considered a trait (expression trait or e-trait). eQTL studies of the model plant *Arabidopsis thaliana* (Decook et al., 2006; Kliebenstein et al., 2006; Keurentjes et al., 2007; West et al., 2007; Burow et al., 2009; Jimenez-Gomez et al., 2010; Cubillos et al., 2012b; Lowry et al., 2013), of crops (Potokina et al., 2008; Swanson-Wagner et al., 2009; Chen et al., 2010; Holloway et al., 2011; Bolon et al., 2014; Wang Y. et al., 2014; Ranjan et al., 2016), and of trees (Drost et al., 2010, 2015) mapped expression variations to both *cis*- and *trans*-acting regulatory polymorphisms: *cis*-eQTL (DNA polymorphisms within or close to the gene) and *trans*-eQTL (polymorphisms located anywhere in the genome). In rice, Wang et al. (2010) performed eQTL mapping of recombinant inbred lines (RILs) derived from two *indica* varieties, Zhenshan 97 and Minghui 63, to identify gene regulatory networks that contribute to an agriculturally important trait. They subsequently developed a new framework that integrates eQTL- and co-expression analysis to narrow the pool of candidate causal genes (Wang J. et al., 2014). An approach to integrate genetic and genomic data, called genetical genomics (Jansen and Nap, 2001), would be useful for identifying possible candidate genes and molecular regulatory mechanisms that play a role in the rapid internode elongation seen in deepwater rice as the water level rises. Gene expression profiling using structured populations segregated into RILs and derived from non-deepwater- and deepwater rice may yield additional insights into the etiology of deepwater responses.

To elucidate the genetic and molecular basis controlling this important trait, we performed microarray experiments in our eQTL analysis of the shoot samples from RIL populations of non-deepwater [Taichung 65 (T65)]- and deepwater rice (Bhadua) subjected to submergence. We identified 10,047 significant eQTLs and significant *trans*-eQTL hotspots on chromosomes 1, 4, and 12 that regulate variations in transcript abundance and exert strong effects on gene expression levels. We also evaluated the QTL for phenotypic traits (phQTL) of the same line. We discuss candidate genes located in the novel eQTL hotspots and candidate target genes of SK1/2. This study provides a useful resource for reconstructing the transcriptional regulatory network at the system-level, and for cloning genes and regulatory control mechanisms that underlie deepwater responses.

## Materials and Methods

### Plant Materials and Growth Conditions

Rice RILs from a cross between Taichung 65 (*O. sativa* L. ssp. *japonica*), and Bhadua (*O. sativa* L. ssp. *indica*; Bangladesh) were used in this study. Seed stocks for the RILs were provided by the National Bio-Resource Project (NBRP) in Japan. We used 85 RILs described by Nagai et al. (2014). The rice seeds were sterilized by boiling at 60°C for 10 min, allowed to germinate in water (30°C for 72 h), and then sown in June 2011 in perforated plastic pots (diameter 10 cm, height 12 cm) filled with soil. They were grown in the greenhouse under a natural light environment. During growth, the water level was beneath the soil surface (air conditions). One month after germination, eight-leaf-stage plants were put into the tank of 160 cm height and completely submerged in water for phenotypic evaluation or microarray analysis.

### Phenotypic Evaluation of Total Internode Length, Plant Height, and Number of Elongated Internodes

Quantitative evaluation of the deepwater response of all RILs and their parental lines was as described elsewhere (Hattori et al., 2009; Nagai et al., 2014). Seven days after submergence treatment, we measured the total internode length (TIL), defined as the length between the uppermost and the basal node, the plant height (PH), and the number of elongated internodes (NEI) in the RILs (Hattori et al., 2009; Nagai et al., 2014) (**Supplementary Figure S1A**). The main culms of each plant were cut along the midline and the TIL was recorded. The NEI was defined as the number of internodes longer than 5 mm. The phenotype of each line was based on the average of no fewer than three plants.

### Genotyping with Illumina’s BeadArray/BeadChip Technology

Genomic DNA was extracted from 85 individuals of the T65/Bhadua RILs and their parental lines using the isoplant method (Zhu et al., 1993) to create a linkage map (Nagai et al., 2014). Briefly, the extracted DNA samples were subjected to genotyping with single-nucleotide polymorphism (SNP) markers using an Affymetrix customized SNP array for rice (Kurokawa et al., 2016). SNPs were detected with the Golden Gate assay of BeadXpress (Illumina, Inc., San Diego, CA, United States). To build the linkage map we used MAPMAKER/EXP version 3.0 (Lander et al., 1987). Our RIL population was genotyped for 188 markers as shown in **Supplementary Figure S2**.

### Microarray Analysis

We used the Agilent Rice Oligo Microarray (44k, custom-made; Agilent Technologies, Redwood City, CA, United States) with the one-color method (Yano et al., 2012). The design of the microarray platform was based on the curated annotation of the rice genome (Sakai et al., 2013) and contained, in addition to four probe-sets corresponding to *SK1* and *SK2* genes, an additional 56 probe-sets corresponding to hormone-related genes and micro RNAs. Since key genes for submergence response are drastically induced until 6 h after submergence treatment (Fukao et al., 2006; Xu et al., 2006; Hattori et al., 2009), we performed the microarray analysis with samples including elongating internodes extracted 6 h after submergence (**Supplementary Figure S1B**). After submergence treatment, the leaves were removed and total RNA was isolated from the region between 1 cm upper part from the uppermost and the basal node in the main culms using the RNeasy Plant Mini Kit (Qiagen, Valencia, CA, United States). Fluorescent probe labeling was with the Agilent Quick Amp Labeling Kit. Labeled cRNA was then fragmented and hybridized on 4 × 44k microarray slides using the Agilent Gene Expression Hybridization Kit in a hybridization oven (65°C for 17 h). All microarray slides were then scanned on an Agilent DNA microarray scanner (model G2505B with G2565BA). Scanned data were analyzed using the default settings of Feature Extraction software (Agilent Technologies). We used unreplicated data for each RIL. Annotation information was from the Rice Annotation Project Database (RAP-DB) (Sakai et al., 2013). All microarray data are in the Gene Expression Omnibus (GEO) (Barrett et al., 2013) and accessible via GEO series accession number GSE87702. Analysis of the microarray data was with R^1^ and the Bioconductor (Gentleman et al., 2004). The Agi4X44PreProcess package^2^ was used for pre-processing and normalization of the microarray data. For quality control and visualization we compared the box plots for raw intensity and background-corrected processed signals; this yielded proper normalized data (**Supplementary Figure S3A**). Using the annotation of probe-sets and rice gene annotation, multiple probe-set identifiers associated with a single gene were summarized to obtain a gene identifier (RAP Id) for the determination of the number of unique eQTLs. The expression value of a gene was averaged across multiple probe-sets mapped to the same gene.

### eQTL and phQTL Mapping

For eQTL mapping, we performed standard quality control, pre-processing of multiple probe-sets assigned to a single gene, and variance filtering. We used 12,264 e-traits in further eQTL mapping to increase the statistical power (Bourgon et al., 2010). Normalized microarray data were used for eQTL mapping with the R package “eqtl” (Cubillos et al., 2012b). The package provides, for example, functions to automatically detect QTLs with supporting intervals from the results of interval mapping for the classification of *cis*- and *trans* acting eQTLs, and for the visualization of the genome-wide relationship between e-traits and eQTLs. Interval mapping was calculated by Haley-Knott regression (Haley and Knott, 1992) implemented in the R/qtl package (Arends et al., 2010). We classified the acting type of eQTLs using the R/eqtl::classify.qtl() function. The function estimates whether an eQTL is *cis*- or *trans*-acting entirely based on logarithm of odds (LOD) support interval definition. The *trans*-eQTLs were defined as those which do not co-localize the affected gene. The *cis*-acting eQTL was defined as those which contains the regulated gene within their LOD support interval. The support interval (si) was estimated by the accepted drop of LOD score from the LOD peak. In this study, we used si = 1.5, default settings in R/eqtl::define.peak() function to define QTLs with support interval.

For phQTL mapping, we used the R package “qtl” (Arends et al., 2010). LOD thresholds were obtained using 1,000 permutations for a significance level of 5%. A global permutation threshold (GPT) was used for determining a genome-wide threshold for statistically significant eQTLs. The significance level for the GPT was set at 0.05. To identify eQTL hotspots, clusters of e-traits co-mapping to the same genomic position, we used the R package “qtlhot” (Neto et al., 2012), which is based on quantile-based permutations.

### Statistical Data Analysis

To identify differentially expressed genes (DEGs) between deepwater and non-deepwater phenotypes, we calculated log_2_-mean expression fold-change of each gene in deepwater type (TIL ≥ 20 cm; Bhadua and 44 RILs) compared to that of non-deepwater type (TIL < 20 cm; T65 and 41 RILs). For the calculation, we used the R package LIMMA (Smyth, 2005); it features false discovery rate (FDR) correction for multiple testing (Benjamini and Hochberg, 1995). Hierarchical cluster analyses (HCAs) and visualization of the expression level (log_2_ ratio) were performed in R. Euclidean distances and the complete linkage method were used for HCA. Principal component analysis (PCA) was applied for the expression data matrix (log_10_-transformed) with autoscaling using SIMCA-P + (v 14.0, Umetrics, Sweden) software. Gene Ontology (GO) term enrichment analysis was with BiNGO software (Maere et al., 2005) with Benjamini and Hochberg (1995) correction for multiple testing (FDR < 0.05).

### Promoter Enrichment Analysis

Our promoter enrichment analysis was based on R-scripts called MotifEnrichment^3^ (Ichihashi et al., 2014; Chitwood et al., 2015). We obtained the 1,000 basepairs upstream of the ATG translational start site of genes with significant *trans*-eQTL hotspots on chromosomes 1, 4, and 12 using the Bioconductor package “BiomaRt” (Durinck et al., 2005, 2009). We employed 100 curated motifs (filename: element_name_and_motif_IUPACsupp.txt) that are based on the Gene Regulatory Information Server AtTFDB database^4^ (Yilmaz et al., 2011). The abundance of motif elements in promoters of genes with *trans*-eQTLs was compared with that of all considered genes using Fisher’s exact test and the Biostrings package (Pages et al., 2016).

### Visualization of phQTLs and Transcript Profiling in a Chromosomal Context

We employed the customized version of QTLVizR^5^ for the visual inspection of phQTLs and for transcript profiling in a chromosomal context. The tool is a web-based application software originally developed by Maloof Lab at the University of California at Davis. The implementation is based on R/shiny^6^; it renders visualization highly dynamic. All data and R/shiny scripts are available upon request to the authors.

## Results

### Phenotypic Evaluation and Transcriptome Analysis

**FIGURE 1:**
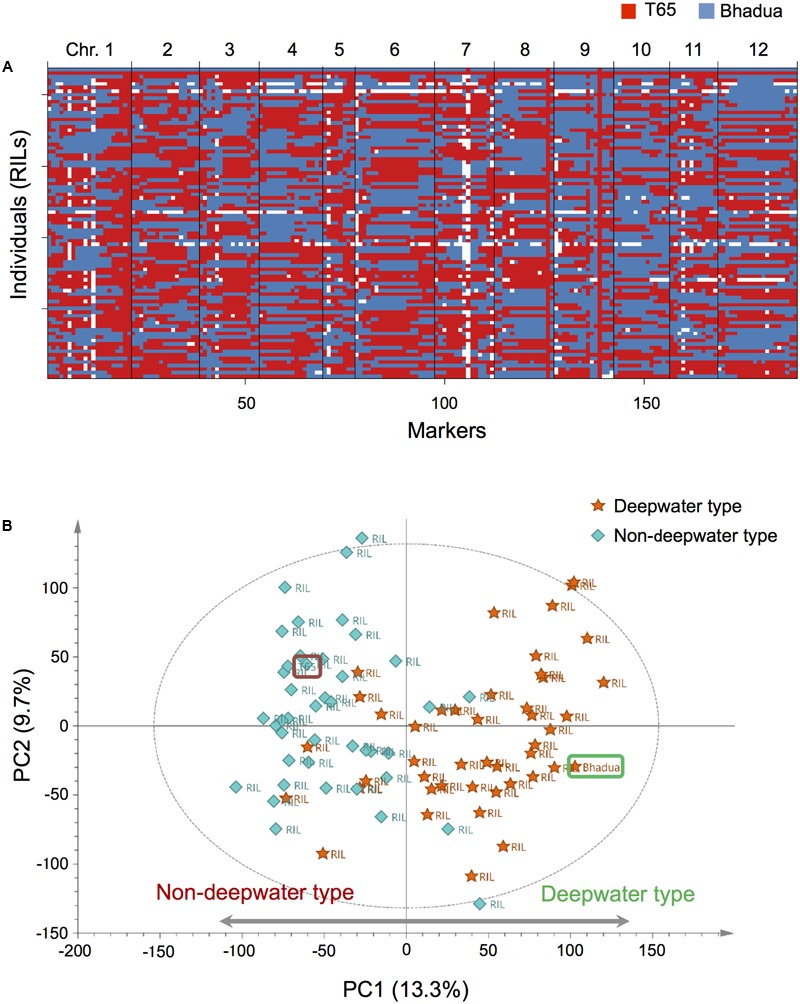
Overview of our genetic and transcriptomic data. **(A)** Haplotypes of 85 recombinant inbred lines (RILs) and their parental lines, T65, and Bhadua. Each row indicates an individual plant, columns correspond to one of the bins (188 markers) arranged in physical order based on the rice chromosomes [Os-Nipponbare-Reference-IRGSP-1.0 (Sakai et al., 2013)]. Red and blue identify the T65 and the Bhadua genotype, respectively. The white box represents missing genotypes. **(B)** Principal component analysis (PCA) of the expression data matrix of the population. The score scatter plot reveals a clear separation between individuals; the first principal component (PC1) accounts for 13.3% of the total explained variance. Orange stars and sky blue diamonds represent RILs with deepwater phenotype (TIL ≥ 20 cm) and non-deepwater phenotype (TIL < 20 cm), respectively. Brown and green rounded squares indicate T65 and Bhadua, respectively. The ellipse indicates Hotelling’s T^2^, with 95% confidence in the score scatter plots.

We performed phenotype analysis of the internode elongation under submergence and genome-wide transcript profiling of the internodes in the parents and 85 RILs, crosses between T65 (non-deepwater rice) and Bhadua (deepwater rice). First, we carried out phenotypic evaluation of TIL, PH, and NEI in the RILs (**Supplementary Figure S1A**). Based on 188 genetic markers (**Supplementary Figure S2**), the haplotypes and the physical genome position of the marker recombination bins for all RILs were investigated (**Figure 1A**). Then, we evaluated phQTLs using quantitative phenotype and haplotype data from the RILs (**Supplementary Figures S4, S5**). Composite interval mapping detected only two significant phQTLs for TIL and PH (LOD = 6.0 at pseudo-marker position “c1.loc40” on chromosome 1 and 11.4 at marker position “ad12009568” on chromosome 12) (**Supplementary Figures S5A,B**). Two significant phQTLs for NEI were located at marker position “P0770_1” on chromosome 1; on chromosome 12 they were at the same position for TIL and PH (**Supplementary Figure S5C**). The location of these two phQTLs for all phenotypic traits was consistent with the QTLs with respect to the internode elongation previously detected by mapping with T65/Bhadua (Kawano et al., 2008) and T65/C9285 (Hattori et al., 2007, 2008).

To assess the biological consistency of different microarray datasets from the same populations we applied PCA of the expression data matrix after appropriate pre-processing and normalization (**Figure 1B** and **Supplementary Figure S3**). The score scatter plot from PCA revealed a clear separation between T65- and Bhadua samples; the first principal component (PC1) accounted for 13.3% of the total explained variance. The plot of RILs indicates that the coordinates in non-deepwater type (TIL < 20 cm) and deepwater type (TIL ≥ 20 cm) are located opposite (**Figure 1B**). These results suggest that global expression pattern of deepwater rice is different from that of non-deepwater rice.

### Identification and Genome-Wide Distribution of *cis-* and *trans*-eQTLs

**FIGURE 2:**
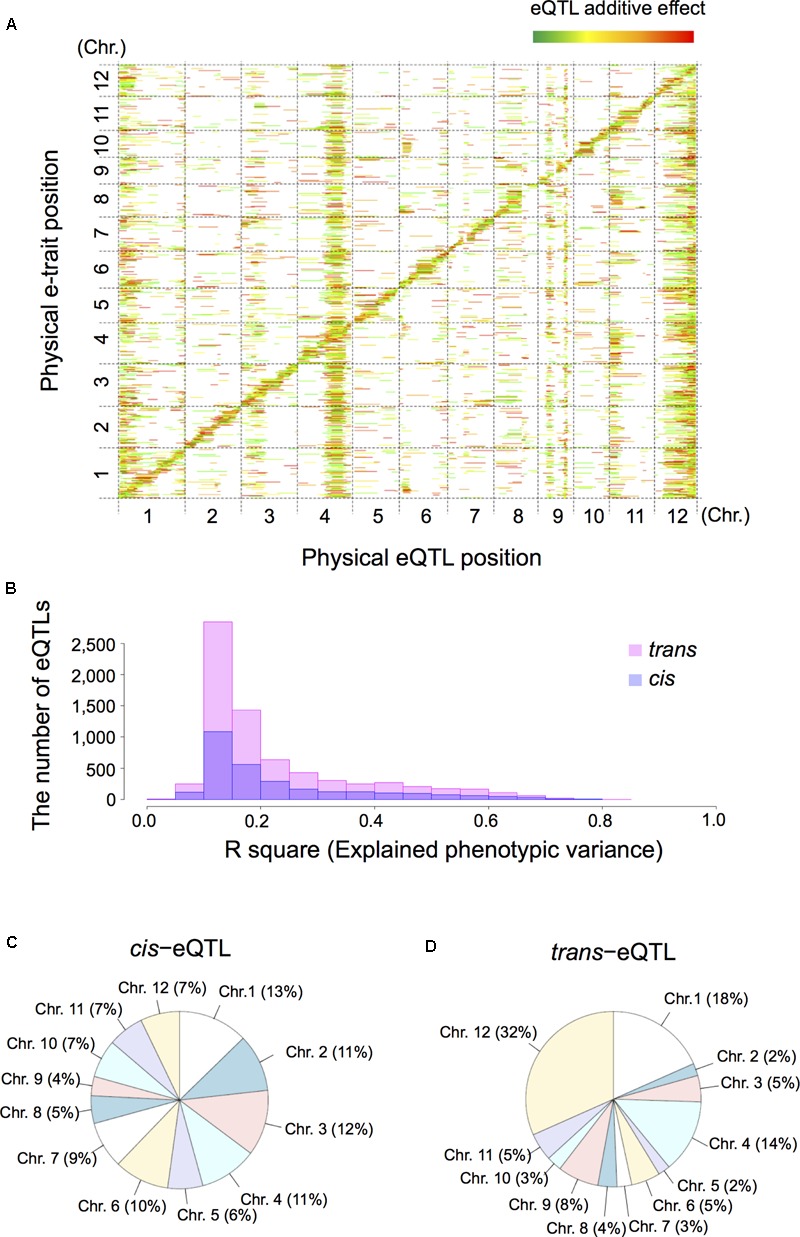
Summary of expression quantitative trait loci (eQTLs) and their location in the rice genome. We analyzed shoot tissue of germinating RIL seeds obtained by crossing T65 and Bhadua rice. **(A)** The eQTL heatmap for T65/Bhadua populations was significant at a 5% false discovery rate (FDR). The *x*-axis represents the genetic position of the detected eQTLs and the *y*-axis the physical position of expressed genes (e-traits). The 12 rice chromosomes are separated by dashed lines. The pseudo-colors indicate the LOD scores (from green to red in order of the increasing LOD score). The bar length along the *x*-axis indicates the eQTL support interval. **(B)** Distribution of the R-square based on analysis of variance (ANOVA), the proportion of phenotypic variation explained by the segregation of an individual QTL or the significant QTL interactions for all eQTLs. **(C)** Pie chart of the proportion of *cis*-eQTLs distributed on 12 rice chromosomes. **(D)** Pie chart of the *trans*-eQTLs distributed on 12 rice chromosomes.

Next, we extracted 12,264 e-traits from genome-wide transcript data by pre-processing and normalization. For eQTL mapping we applied interval mapping [Haley-Knott regression (Haley and Knott, 1992)] with R/qtl (Arends et al., 2010) and R/eqtl packages (Cubillos et al., 2012b). Subsequent mapping identified 10,047 significant eQTLs (FDR < 0.05) comprised of 2,902 *cis*-eQTLs (28.9%) and 7,145 *trans*-eQTLs (71.1%); 7,078 e-traits featured at least one eQTL (Supplementary Table S1). To determine the number of eQTLs for each e-trait we examined the genome-wide relationship between e-traits and eQTLs on a heatmap. This examination was based on the physical positions of the markers and their corresponding e-traits on the 12 rice chromosomes (**Figure 2A**). We also estimated the phenotypic contribution with the R-square based on analysis of variance (ANOVA) for individual eQTLs and their significant interactions for each e-trait (**Figure 2B**). We defined eQTLs based on a threshold of LOD significance calculated by permuting the phenotypes while maintaining the genotype across the RILs and a 1.5 LOD drop-support interval. On the 12 rice chromosomes the proportion of *cis*-eQTLs (**Figure 2C**) was homogeneously while the proportion of *trans*-eQTLs (**Figure 2D**) was heterogeneously distributed, especially on chromosomes 1 (18% of *trans*-eQTLs), 4 (14%), 9 (8%), and 12 (32%).

### Detection of *cis*-eQTLs for SK1/2

**FIGURE 3:**
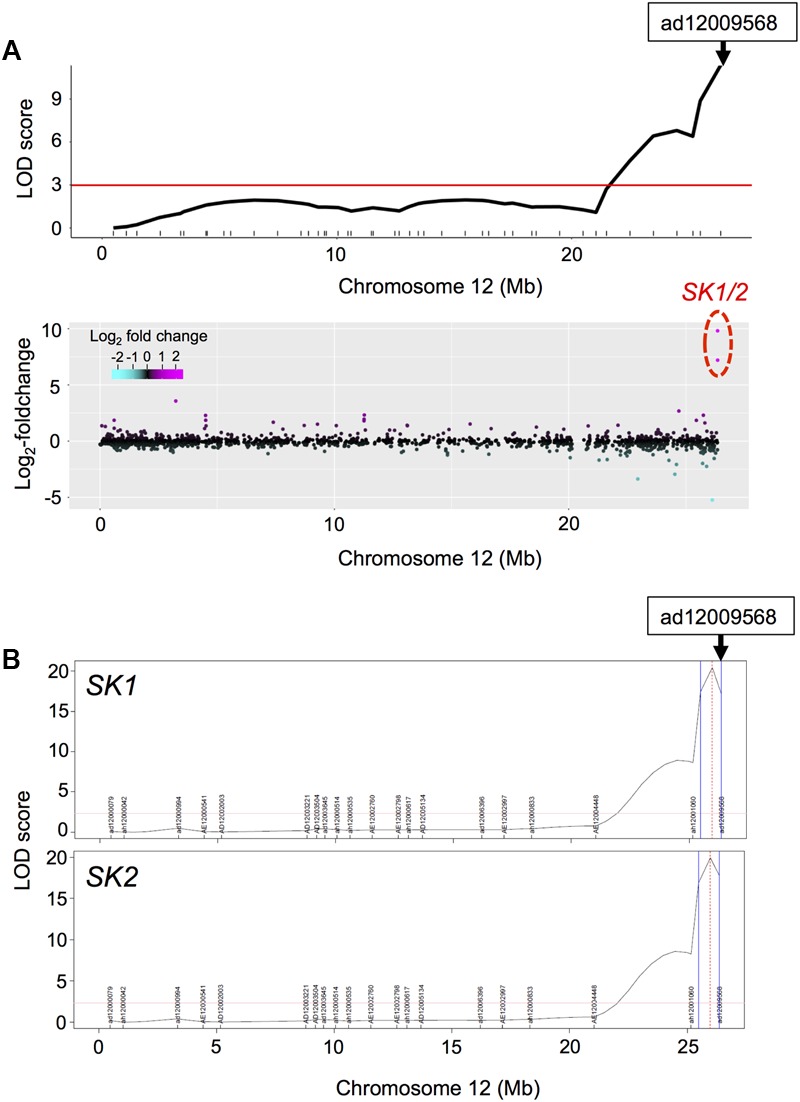
Phenotypic QTL (phQTL) and eQTL for *SK1*/*2*. **(A)** LOD statistics of phQTLs for total internode length (TIL) on chromosome 12 are shown (upper panel). The differential expressions between non-deepwater- and deepwater-type RILs are plotted along the physical genome position in log_2_-fold change (bottom panel). Note that transcription factors SK1 and SK2 exist in Bhadua but not in T65 plants. The red horizontal line indicates LOD = 3. **(B)** LOD statistics of eQTLs for *SK1*/*2* gene expressions. Vertical blue lines represent support interval for the detected QTLs.

As described above, we detected phQTLs on end of chromosome 12 (**Supplementary Figure S5**), whose responsible gene would be the *SK1/2* (Hattori et al., 2009). We next visualized LOD statistics of the phQTLs for TIL (**Figure 3A**, upper panel) and the DEGs, calculated from log_2_-fold-change between expressions of RILs with deepwater phenotype (TIL ≥ 20 cm) and non-deepwater phenotype (TIL < 20 cm) (**Figure 3A**, lower panel) along the physical genomic position on chromosome 12. The location of *SK1*/*2* genes was consistent with that of phQTLs for TIL (**Figure 3A**). The peak of eQTLs for the expression of *SK1*/*2* was detected as *cis*-eQTL in the region where their own genes were located (**Figure 3B**). Thus, the responsible genes of the *cis*-eQTL are at least *SK1*/*2* themselves because these genes exist in Bhadua but not in T65 (Hattori et al., 2009).

### The Identification of Significant eQTL Hotspots Revealed Novel Genomic Loci That May Be Responsible For Expression Variations between Non-deepwater- and Deepwater Rice

**FIGURE 4:**
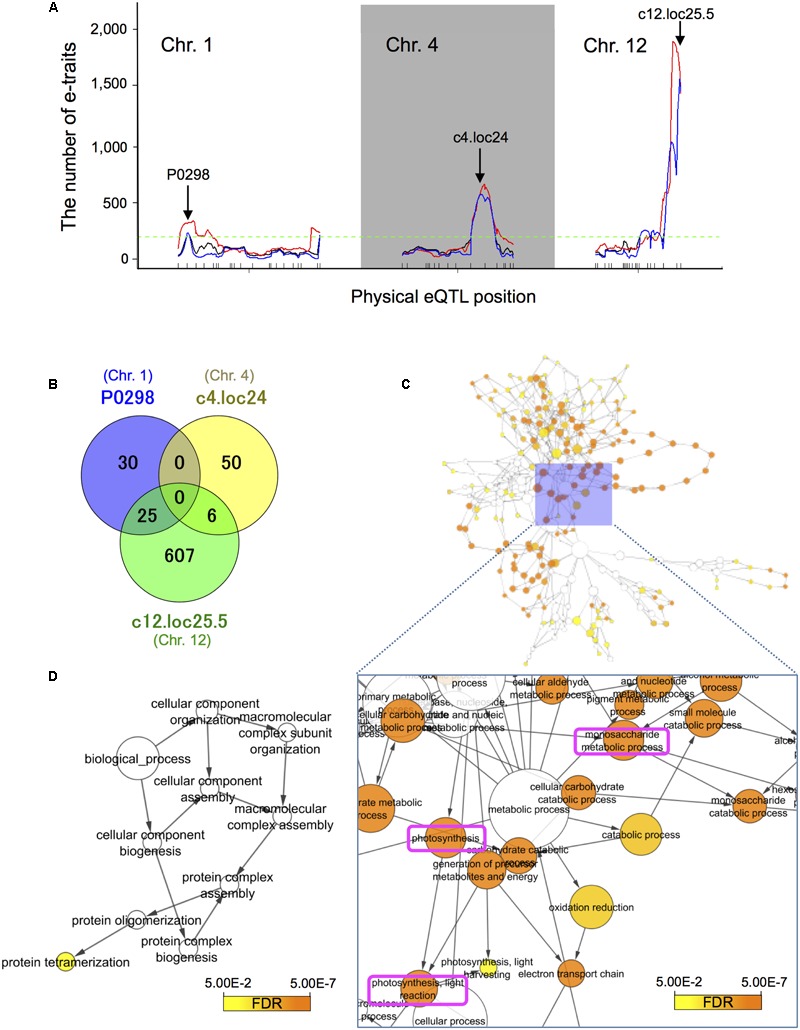
Expression quantitative trait loci hotspot size significance profile and functional assessment of genes with *trans*-eQTLs. **(A)** The major significant eQTL hotspots were derived with a quantile-based permutation approach (Neto et al., 2012). All permutation thresholds were calculated targeting a genome-wide error rate (GWER) < 0.05. The major hotspots on chromosomes 1, 4, and 12 were identified. Arrows indicate the (pseudo-)marker name of the maximum LOD peak. Black lines represent raw eQTL counts, red identifies counts smoothed over a 5 cM window, and blue the sliding LOD threshold approach. The horizontal dashed green line indicates 5% threshold of 195 that is for the number of e-traits with significant LOD at a position (Neto et al., 2012). **(B)** Venn diagram of the number of e-traits with *trans*-eQTL at the physical position of the peaks within the three eQTL hotspots. **(C)** Over-represented gene ontology (GO) terms among 638 genes with *trans*-eQTLs at physical pseudo-position “c12.loc25.5” on chromosome 12. We used BiNGO (Maere et al., 2005) (FDR < 0.05). The top three significantly enriched terms were “photosynthesis” (FDR = 8.8E-33), “photosynthesis, light reaction” (FDR = 1.3E-28), and “monosaccharide metabolic process” (FDR = 1.1E-20). **(D)** Over-represented GO terms among 55 genes with *trans*-eQTLs at physical position “P0298” on chromosome 1. The term “protein tetramerization” (GO-Id: 51262) was significantly enriched among the genes (FDR = 2.9E-2). Note that there are no significant GO terms in the analysis for genes with *trans*-eQTLs at physical pseudo-position “c4.loc24” on chromosome 4.

Groups of e-traits that co-map to the same genomic region are called eQTL hotspots (Brem et al., 2002; Schadt et al., 2003; Breitling et al., 2008). They are biologically meaningful because they may harbor key regulators. We identified the major significant eQTL hotspots using a quantile-based permutation approach called the NL-method (Neto et al., 2012); all permutation thresholds were calculated targeting a genome-wide error rate (GWER) < 0.05. This analysis revealed the major eQTL hotspots on chromosomes 1, 4, and 12 (**Figure 4A** and **Supplementary Figure S6**); some of their positions on these chromosomes are enriched for *trans*-eQTLs. The eQTL hotspot at pseudo-marker position “c12.loc25.5” on chromosome 12 was consistent with the location of *SK1*/*2*. The eQTL hotspots at “P0298” on chromosomes 1 and “c4.loc24” on chromosome 4 located at different position from phQTLs detected in this study (**Supplementary Figure S5**) and the three major phQTLs reported by others (Tang et al., 2005; Hattori et al., 2007, 2008; Kawano et al., 2008).

### Candidate Target Genes of the Novel eQTL Hotspots

The hypothesis that the candidate target genes of the eQTL hotspots harbor *trans*-eQTLs at the physical position of each eQTL hotspot provides a clue for a potential regulatory network where the eQTL hotspots control changes in the transcript abundance of numerous genes in *trans*. This may result in phenotypic variations. First, we narrowed the pool down to 55, 56, and 638 e-traits that feature *trans*-eQTL at physical position “P0298” on chromosome 1, “c4.loc24” on chromosome 4, and “c12.loc25.5” on chromosome 12, respectively (Supplementary Table S2). Next, from these e-traits, we searched for DEGs, between expressions of deepwater phenotype and non-deepwater phenotype on physical position of RILs for each e-traits. We found that 23.6% (13/55 genes) of e-traits with *trans*-eQTL at “P0298,” 10.7% (6/56 genes) of e-traits with *trans*-eQTL at “c4.loc24,” and 30.1% (192/638 genes) of e-traits with *trans*-eQTL at “c12.loc25.5” appeared in groups of significantly up-regulated DEGs (Supplementary Table S2).

Analysis of the relationships among the three groups of e-traits at different physical positions revealed no e-trait sharing *trans*-eQTLs at “P0298” and “c4.loc24” (**Figure 4B**). On the other hand, 45.5% (25/55 genes) of e-traits with *trans*-eQTL at “P0298” and 10.7% (6/56 genes) of e-traits with *trans*-eQTL at “c4.loc24” are shared with *trans*-eQTLs at position “c12.loc25.5.” To assess whether the eQTL hotspots are associated with biological functions, we conducted GO (The Gene Ontology Consortium, 2014) term enrichment analysis of genes with *trans*-eQTL at physical (pseudo-)position “c12.loc25.5” on chromosome 12 and at “P0298” on chromosome 1 using BiNGO (Maere et al., 2005) (FDR < 0.05). The three top significantly enriched terms among genes with *trans*-eQTLs at physical pseudo-position “c12.loc25.5” on chromosome 12 were “photosynthesis” (FDR = 8.8E-33), “photosynthesis, light reaction” (FDR = 1.3E-28), and “monosaccharide metabolic process” (FDR = 1.1E-20) (**Figure 4C** and Supplementary Table S3B), indicating potential SK1/2 target genes. The term “protein tetramerization” (GO-Id: 51262) was significantly enriched among genes with *trans*-eQTLs at physical positions on chromosome 1 (FDR = 2.9E-2) (**Figure 4D** and Supplementary Table S3A). As for physical pseudo-position “c4.loc24” on chromosome 4, our analysis for genes with *trans*-eQTLs revealed no significant GO terms.

### Candidate Causal Genes Located in the Novel eQTL Hotspots on Chromosomes 1 and 4

**FIGURE 5:**
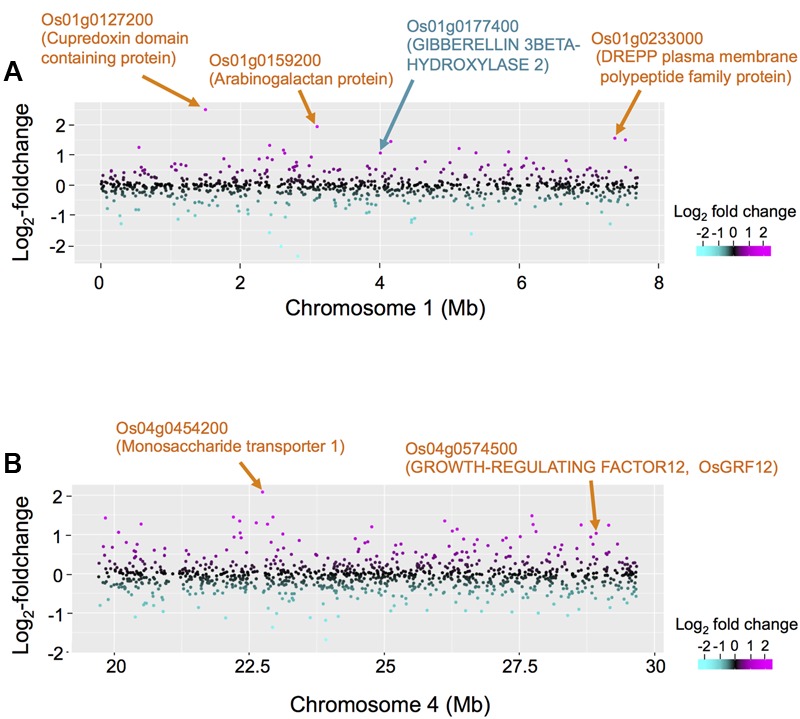
Comparison between differentially expressed genes (DEGs) and eQTL hotspots on chromosomes 1 and 4. **(A)** The differential expressions in non-deepwater- and deepwater-type RILs are plotted on the region of eQTL hotspots on chromosome 1 (genome position: 0–7.72 Mb) in log_2_-foldchanges. The red horizontal line indicates LOD = 3. **(B)** The differential expressions in non-deepwater- and deepwater-type RILs are plotted on the region of eQTL hotspots on chromosome 4 (genome position: 19.7–29.7 Mb) along the physical genome position in log_2_-foldchange. The red horizontal line indicates LOD = 3.

To obtain insights into the novel eQTL hotspot on chromosome 1 we identified 18 DEGs within ±5 cM of the eQTL hotspot located on chromosome 1 (Supplementary Table S4). Up-regulated DEGs included genes encoding the cupredoxin domain containing protein, arabinogalactan, the DREPP plasma membrane polypeptide family protein, and gibberellin 3β-hydroxylase (DWARF 18; D18) (**Figure 5A**). We also identified 27 DEGs within ±5 cM of the eQTL hotspot located on chromosome 4 (Supplementary Table S4). The five top ranking up-regulated genes with large fold-changes encoded monosaccharide transporter 1 (**Figure 5B**), the major sperm protein domain containing protein, glyceraldehyde 3-phosphate dehydrogenase, the plant invertase/pectinesterase inhibitor domain containing protein, and heavy metal transport/detoxification protein domain containing protein. We also identified a gene encoding *O. sativa* GROWTH-REGULATING FACTOR 12 (OsGRF12) as a significantly up-regulated DEG (see “Discussion”). Our list contained six down-regulated genes, including genes encoding phragmoplast-associated kinesin-related protein 1 and protein kinase- and ubiquitin domain containing proteins.

**FIGURE 6:**
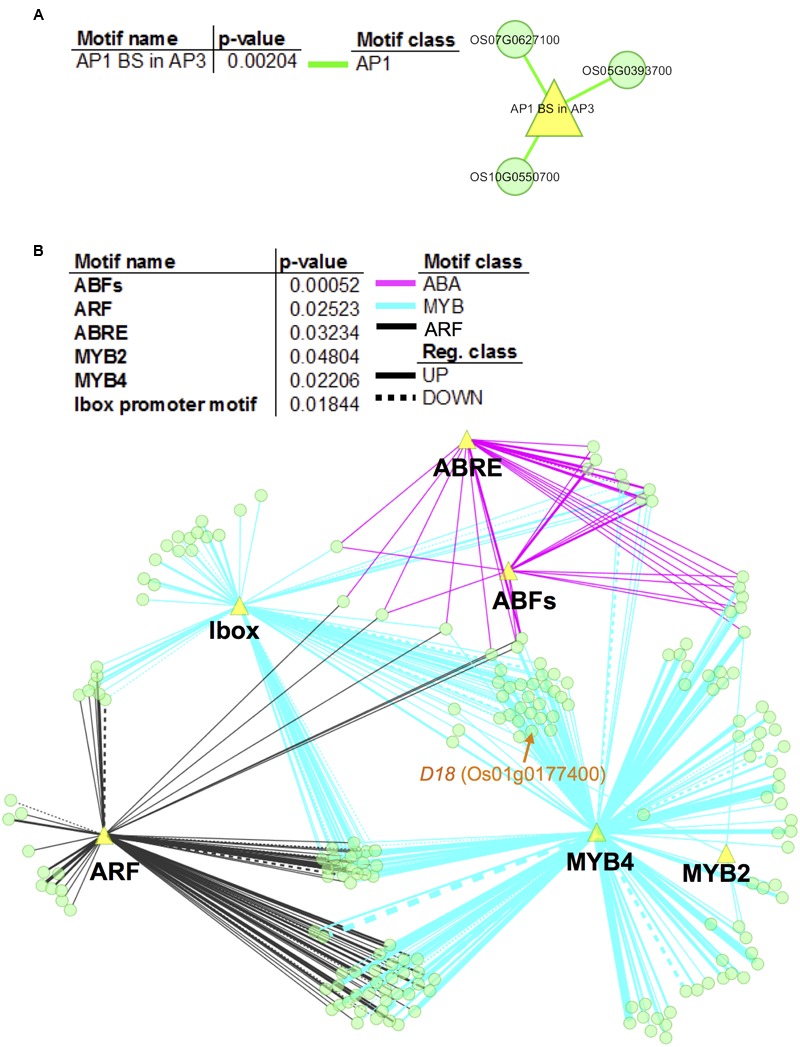
Network visualization of enriched motifs and genes with *trans*-eQTLs hotspots. We performed promoter enrichment analysis for promoters of e-traits that feature *trans*-eQTL at physical position “c4.loc24” on chromosome 4 **(A)** and “c12.loc25.5” on chromosome 12 **(B)**. Significantly enriched motifs were listed in the tables (*p* < 0.05). The enrichment of different types of motif is visualized in colors representing the motif class. The network represents the relationship between evaluated genes and the enriched motifs (yellow triangles). Network nodes (green) indicate genes. Motif classes are as follows: yellow green, AP1; magenta, ABA; black, ARF; and cyan, MYB. Edge thickness is proportional to the number of motifs that exist in the promoters of the respective genes. Note that the analysis showed no significant motifs in 55 e-traits on chromosome 1.

To further elucidate the gene regulatory networks that underlie expression variations between non-deepwater- and deepwater rice, promoters of the candidate target genes of the novel eQTL hotspots were investigated for over-represented motifs. Our analysis identified significant enrichment (Fisher’s exact test, *p* < 0.05) for an AP1 binding site (consensus sequence CCATTTTTAG) for candidate genes with *trans*-eQTLs at a physical position on chromosome 4 (**Figure 6A**); there were no significant motifs in 55 e-traits on chromosome 1. As for the promoters of candidate genes with *trans*-eQTL on chromosome 12, the enriched motifs were involved in ABA signaling including ABRE (ABA responsive element)- and ABF (ABRE-BINDING FACTOR) binding sites (**Figure 6B**). The second largest class of enriched motifs was related to MYB transcription factors, especially the MYB2 binding site motif [TAACT(G/C)GTT] and the MYB4 binding site motif [A(A/C)C(A/T)A(A/C)C]; the other enriched motif was the ARF (Auxin Response Factor) binding site motif (TGTCTC). Our observation suggests that these transcription factors play a role in the promotion of rapid internode elongation in deepwater rice.

## Discussion

### Identification of eQTLs Controlling Gene Expression Networks Underlying Deepwater Responses

Transcript abundance, used as e-traits in genetical genomics studies, is highly heritable and genetically controlled (Jansen and Nap, 2001; Kliebenstein, 2009; Druka et al., 2010; Cubillos et al., 2012a; Chitwood and Sinha, 2013). Our aim was to map QTLs for gene expression that exist behind the regulatory control mechanisms underlying deepwater responses. Genetical genomics study using internode samples of RIL populations derived from non-deepwater (T65)- and deepwater rice (Bhadua) can be used as a model to gain a better understanding of the genotype-phenotype relationships elicited in response to submergence in monocot plants. Using genetic markers, microarray technology, and RIL population (**Figure 1**), we identified thousands of significant eQTLs (**Figure 2**).

Others (Brem et al., 2002; Schadt et al., 2003; Breitling et al., 2008) applied genetical genomics approaches to identify multiple e-traits that map to the same genomic locus; they are called eQTL hotspots. As such hotspots have been reported in plants such as Arabidopsis, rice, and soybean, it is expected that eQTL hotspots can be used for the identification of key regulators that elicit a wide range of downstream changes in gene expressions. However, the identification of significant biologically meaningful eQTL hotspots is hampered by undesirable variations from non-genetic factors such as uncontrolled environmental conditions and the physiological seed quality [for example, see (Breitling et al., 2008)]. To avoid the detection of spurious eQTL hotspots attributable to non-genetic variations we applied quantile-based permutation thresholds (Neto et al., 2012).

### Detailed Evaluation of the eQTLs and the Novel *trans*-eQTL Hotspots to Infer Causal Regulatory Networks of Deepwater Responses

Our eQTL mapping highlighted that the three eQTL hotspots on chromosomes 1, 4, and 12 were possible candidate factors for internode elongation in deepwater rice (**Figure 4**). The eQTL hotspots on chromosome 12 co-localized with phQTL locating *SK1/2* (**Figures 3, 4**), suggesting that *SK1/2* are one of master regulator genes that controls the deepwater response. No significant phQTLs was observed on the locations of eQTL hotspots on chromosomes 1 and 4 (**Figure 4** and **Supplementary Figure S5**). These inconsistencies could be explained by following two reasons. One reason is that resolution of the QTL analysis was not enough for detection of phQTL on chromosome 1 and 4; we detected small peaks of phQTL on both chromosome 1 and 4 although they were not statistically significant (**Supplementary Figure S5**). Especially, phQTL on chromosome 4 has been detected on QTL analysis for internode elongation focusing on response in early developmental stage (Nagai et al., 2012). Another reason is that these eQTL hotspots may contribute to other responses under submergence rather than internode elongation. Further QTL analysis with higher resolution is needed to evaluate these possibilities. On the other hand, no significant eQTL hotspot was detected at the location of phQTL at c1.loc40 on chromosome 1 (**Figure 4** and **Supplementary Figure S5**). This imply that responsible gene of the phQTL at c1.loc40 could directly function on internode elongation rather than transcriptional regulation.

The *trans*-eQTL enrichment on chromosomes 1, 4, and 12 may indicate the location of a master regulator gene that controls the deepwater response. The *D18* gene was detected as one of the DEGs in the eQTL hotspot on chromosome 1 (**Figure 5A** and Supplementary Table S4). *D18* encodes *O. sativa* gibberellin 3 oxidase 2 (OsGA3ox2) known to control shoot elongation in rice by catalyzing the final step in the synthesis of bioactive gibberellins (GAs) (Itoh et al., 2001). It has been reported to be expressed in elongating internodes (Kaneko et al., 2003), and that *D18* loss-of-function blocks deepwater-dependent internode elongation (Ayano et al., 2014). As the expression of *D18* is significantly higher in deepwater phenotype than non-deepwater phenotype (**Figure 5A**), it is a strong candidate as the regulatory gene of the eQTL hotspot, suggesting that genes responsible for this eQTL hotspot may contribute to the specific gene expression resulting in internode elongation under submergence.

We identified a significant eQTL hotspot on chromosome 4 (**Figure 5B**). As one of the DEGs, we detected *OsGRF12* in the eQTL hotspot on chromosome 4; its expression was higher in deepwater phenotype than non-deepwater phenotype (**Figure 5B** and Supplementary Table S4). Choi et al. (2004) reported that *OsGRF12*, a member of the plant-specific GRF transcriptional regulator family, expressed in internodes containing the intercalary meristem and in a part of the elongation zone, was induced by GA treatment. The overexpression of *OsGRF10*, a member of the GRF family with the highest similarity to *OsGRF12*, led to an increase in the number of internodes; a knock-down of both *OsGRF3* and *OsGRF4* resulted in a decrease in the internode length (Kuijt et al., 2014). Although its physical genome position is not consistent with the peak of phQTLs, it is possible that *OsGRF12* is a candidate gene for the eQTL hotspot and it may play an important role in the transcriptional regulation of internode elongation under plant submergence.

We also looked for the candidate target genes of the three eQTL hotspots by searching for genes with *trans*-eQTLs at the physical position of each eQTL hotspot. Additional GO enrichment analysis of the candidate target genes of the novel eQTL hotspot on chromosome 12, locating *SK1/2*, showed that the GO functional categories photosynthesis, light reaction, monosaccharide metabolic process were significantly over-represented (**Figure 4C**). Thus, it is possible that SK1/2 mainly regulate these GO enriched genes for deepwater-rice specific submergence response. Similar to rice, in dicot plants, two *Rumex* species use ‘escape’ and ‘quiescence’ strategies upon submergence (Van Veen et al., 2013). Genome-wide transcriptome analysis by RNA-seq technology for comparison of the *Rumex* species suggested that light responsive signaling and plant hormone-related genes play a vital role in controlling the underwater elongation response (Van Veen et al., 2013). In our study, these genes were not enriched in the SK1/2 candidate target. Further approaches such as detailed RNA-seq analysis or identification of direct target of SK1/2 are needed to compare molecular mechanism of the submergence response in these monocot and dicot species.

### Enhancement of Our Understanding of SK1/2-Dependent and Independent Pathways

Our results suggest that the downstream regulation of the eQTL hotspots on chromosomes 1 and 4 is independent, and that the target genes are partially regulated by SK1/2 (**Figure 4B**). The *D18* gene, one of the candidate genes for eQTL hotspots on chromosome 1, harbors *trans-*eQTLs on chromosome 12 around the SK1/2 region (Supplementary Table S2C), suggesting that the expression of *D18* is regulated by SK1/2. On the other hand, *OsGRF12*, one of the candidate genes for the eQTL hotspot on chromosome 4, may be expressed under the independent regulation of SK1/2 because it does not harbor *trans*-eQTLs at the physical position of the SK1/2 region (Supplementary Table S2C). Promoter enrichment analysis revealed that ABA, ARF, and MYB-related motifs were enriched in promoters of candidate target genes with the *trans*-eQTL hotspot on chromosome 12 (**Figure 6B**). In the AP2/ERF superfamily, SK1/2 belong to group VII including Arabidopsis hypoxia-responsive transcription factors such as RAP2.2 and RAP2.12 (Hattori et al., 2009). Given that RAP2.2 and RAP2.12 bind to the evolutionarily conserved motif consisting of the MYB binding site and the GC-rich region (Gasch et al., 2016), SK1/2 may transactivate these candidate target genes by binding to motif similar to the MYB-related motifs as well as to ABA and ARF.

**FIGURE 7:**
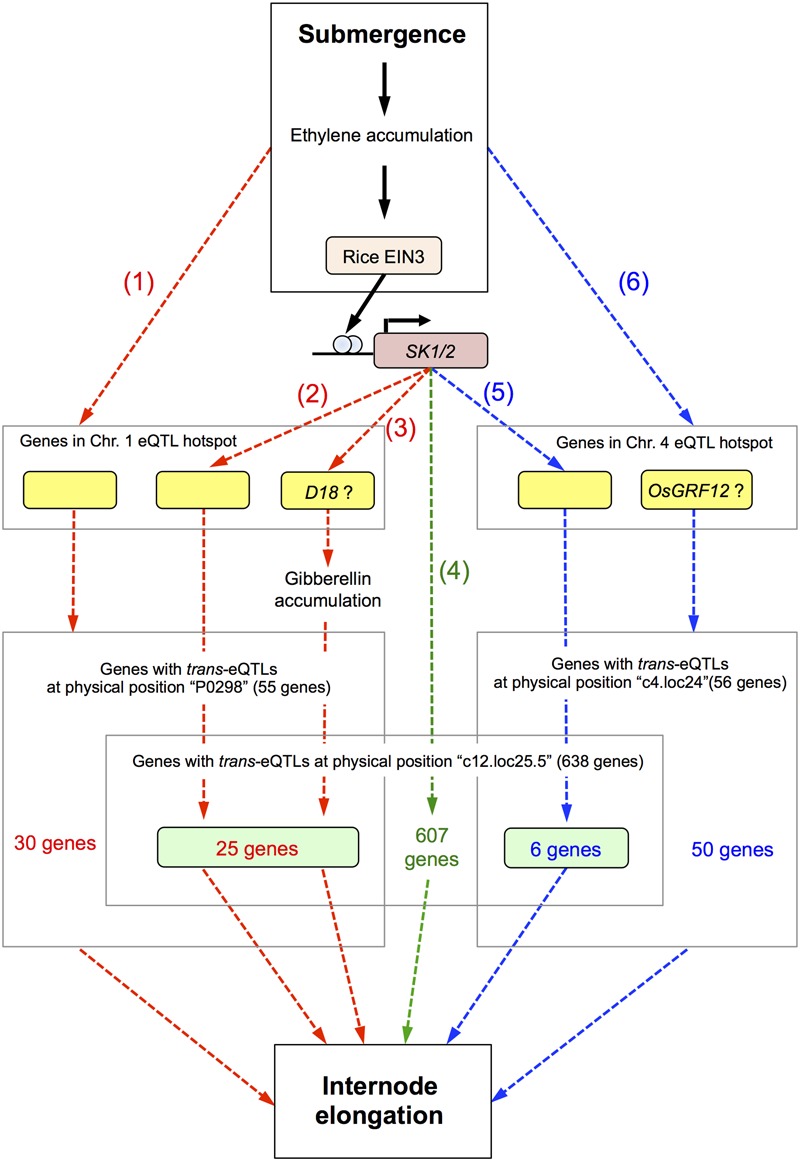
Hypothetical model of signaling for internode elongation in submerged deepwater rice. Bold black arrows represent known pathways and dashed arrows the hypothetical pathways described in this study. Red and blue dashed arrows indicate the pathways regulated by genes in the eQTL hotspot on chromosome 1 and 4, respectively. Green dashed arrows indicate the pathway regulated by SK1/2 independent of the eQTL hotspots on chromosomes 1 and 4. Details are described in the “Discussion.”

Based on our observations, we present a hypothetical model of the signaling pathways leading to internode elongation in deepwater rice in response to submergence (**Figure 7**). When deepwater rice plants are submerged, ethylene accumulates, and this phytohormone induces *SK1*/*2* expression via the rice ETHYLENE-INSENSITIVE 3 (EIN3)-like transcription factor (Hattori et al., 2009). SK1/2 promote the accumulation of GA and/or GA signal transduction, positively regulating elongation of the internodes and leaves. Based on our eQTL mapping findings we hypothesize the presence of 6 pathways for regulating the deepwater response (**Figure 7**).

The first pathway is regulated by genes in the eQTL hotspot on chromosome 1 and triggered by submergence and/or ethylene accumulation. The second is regulated by genes in the eQTL hotspot on chromosome 1 under direct or indirect regulation by SK1/2. The third is regulated by the *D18* gene in the eQTL hotspot on chromosome 1 under direct or indirect regulation by SK1/2. The *D18* gene product may directly regulate an increase in active GA. Since it is reported that the precursor level of active GAs is increased under submergence (Ayano et al., 2014), increase of *D18* gene expression may contribute to acceleration of production of active GA. The fourth pathway is directly or indirectly regulated by SK1/2 and independent of the eQTL hotspots on chromosomes 1 and 4. The candidate 607 genes in this pathway may link between SK1/2 and submergence-induced internode elongation. The fifth is regulated by the gene in the eQTL hotspot on chromosome 4 under direct or indirect regulation by SK1/2. The sixth pathway is regulated by genes in the eQTL hotspot on chromosome 4 and triggered by submergence and/or ethylene accumulation. OsGRF12 may contribute to this pathway as a response gene of the eQTL hotspot on chromosome 4. Detailed molecular analyses such as regulation by GA or ethylene would help whether these candidate genes actually function in these pathways for internode elongation under submergence.

## Conclusion

In this study, we present genome-wide eQTL mapping based on a microarray platform using RIL populations from T65- and Bhadua rice. Our analysis shows the existence of novel *trans*-eQTL hotspots on chromosomes 1, 4, and 12. The identification of *cis*- and *trans*-eQTLs yielded an array of candidate genes ready for further QTL analysis in structured populations with a deepwater response. Further QTL analyses with higher population size, marker density, and transcriptome under time-course submergence treatment will efficiently narrow down candidate genes for deepwater response. The association between *trans*-eQTLs and phQTLs in a given phenotype may provide a clue to candidate genes associated with a specific trait but does not necessarily yield candidates involved in the generation of the phQTLs. Such candidate genes will be useful for gene discovery and for studies on the deepwater response in rice. Integrating other omics data, e.g., proteomics and metabolomics (Fukushima et al., 2009; Fukushima and Kusano, 2014), with eQTL data from the same lines will greatly help to link hundreds of cellular molecules to eQTLs. Systematic and comprehensive studies may make possible the elucidation of complex regulatory networks underlying the genetic architecture of deepwater responses.

## Availability of Supporting Data

All microarray data are available in the NCBI GEO database (Barrett et al., 2013) (accession no. GSE87702).

## Author Contributions

TK, MA, and AF conceived and designed the experiments. TK, KN, YK, and YN performed the experiments. TK, MK, and AF analyzed the data. TK, KN, YK, YN, HY, MA, and AF contributed reagents/materials/analysis tools. TK and AF wrote the paper.

## Conflict of Interest Statement

The authors declare that the research was conducted in the absence of any commercial or financial relationships that could be construed as a potential conflict of interest.

## Footnotes

1 https://cran.r-project.org/

2 http://www.bioconductor.org/packages//2.10/bioc/html/Agi4x44PreProcess.html

3 https://bitbucket.org/jnmaloof/motifenrichment/

4 http://arabidopsis.med.ohio-state.edu/AtcisDB/bindingsites.html

5 https://github.com/tiaho/QTL-Visualization

6 http://shiny.rstudio.com/

## Supplementary Material

FIGURE S1 Schematic view of our experimental setup. Diagram showing how rice plants were submerged for measurement of the TIL and for RNA extraction. **(A)** Eight-leaf-stage plants of the RILs and their parents were grown in pots and completely submerged in water. For phenotype analysis, TIL was measured 7 days after submergence treatment and defined as the length between the uppermost and the basal node. **(B)** For microarray analysis, the RILs and their parents were submerged for 6 h. The region between 1 cm upper part from the uppermost and the basal node was used for RNA extraction. Leaves were removed from their nodes advanced to the extraction. Abbreviations: TIL, total internode length; PH, plant height; NEI, number of elongated internodes; and SAM, shoot apical meristem.

FIGURE S2 Linkage map of T65/Bhadua RILs.

FIGURE S3 Overview of genome-wide transcriptome data. **(A)** Results of normalization of the microarray data for the RILs and their parents. **(B)** Dendrogram of the RILs and their parents based on microarray data. Euclidean distances and the complete linkage method were used for hierarchical clustering.

FIGURE S4 Statistical distribution of TIL, PH, and NEI among the RILs and their parents. **(A)** TIL, **(B)** PH, and **(C)** NEI are shown. White and black arrowheads represent the phenotypic values for T65 and Bhadua rice, respectively. Abbreviations: TIL, total internode length; PH, plant height; NEI, number of elongated internodes.

FIGURE S5 Overview of phQTL data of the RILs. LOD statistics of phQTLs for TIL **(A)**, PH **(B)**, and NEI **(C)**. Arrows point to the pseudo-marker or marker positions. The candidate regions of *qRIE1* and *qRIE12* (Kawano et al., 2008) are shown with blue lines. Abbreviations: TIL, total internode length; PH, plant height; NEI, number of elongated internodes; and RIE, rate of internode elongation. The red horizontal line indicates LOD = 3.

FIGURE S6 Overview of eQTL hotspot size significance profile. The eQTL hotspots in the 12 rice chromosomes were derived with a quantile-based permutation approach (Neto et al., 2012). Data are presented essentially as shown in **Figure 4A**.
