# Supplementary figures and images for: eQTLs Regulating Transcript Variations Associated with Rapid Internode Elongation in Deepwater Rice
Source: Front Plant Sci. 2017 Oct 13;8:1753. doi: 10.3389/fpls.2017.01753 (PMC5645499; doi:10.3389/fpls.2017.01753)

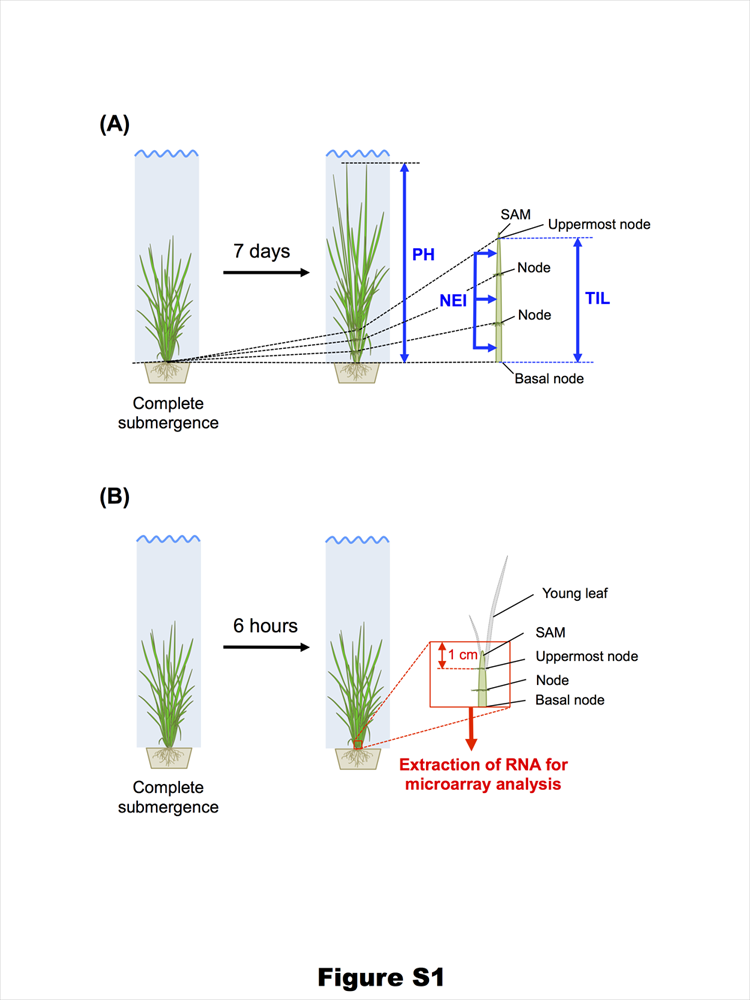

Supplement: FIGURE S1 — Schematic view of our experimental setup. Diagram showing how rice plants were submerged for measurement of the TIL and for RNA extraction. (A) Eight-leaf-stage plants of the RILs and their parents were grown in pots and completely submerged in water. For phenotype analysis, TIL was measured 7 days after submergence treatment and defined as the length between the uppermost and the basal node. (B) For microarray analysis, the RILs and their parents were submerged for 6 h. The region between 1 cm upper part from the uppermost and the basal node was used for RNA extraction. Leaves were removed from their nodes advanced to the extraction. Abbreviations: TIL, total internode length; PH, plant height; NEI, number of elongated internodes; and SAM, shoot apical meristem. [file Image_1.TIFF]

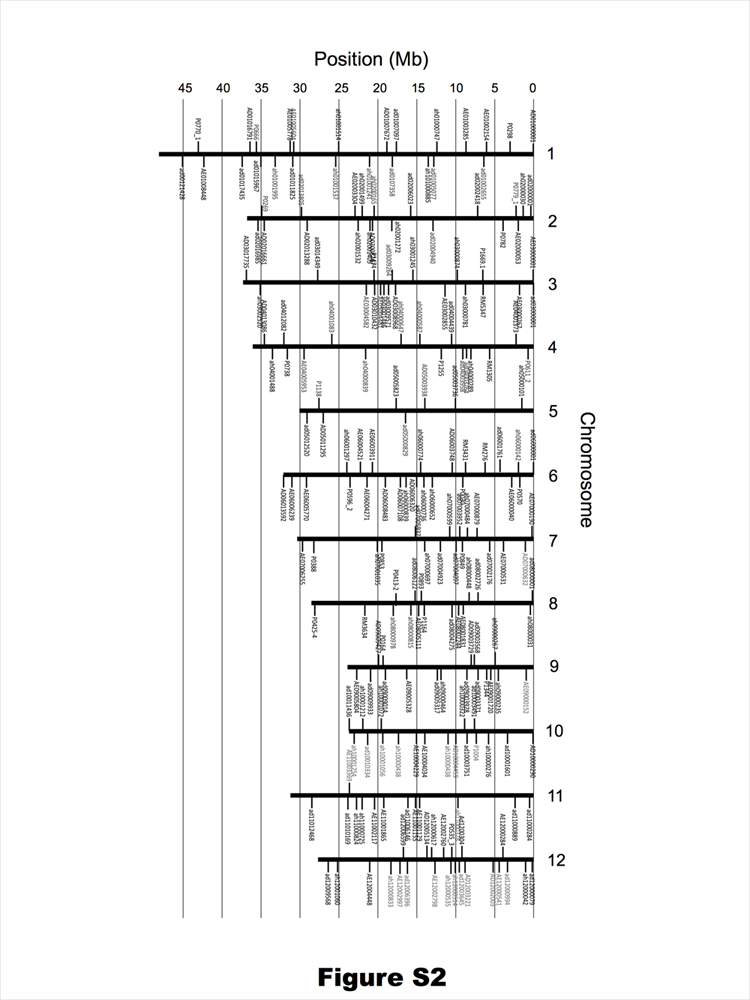

Supplement: FIGURE S2 — Linkage map of T65/Bhadua RILs. [file Image_2.TIFF]

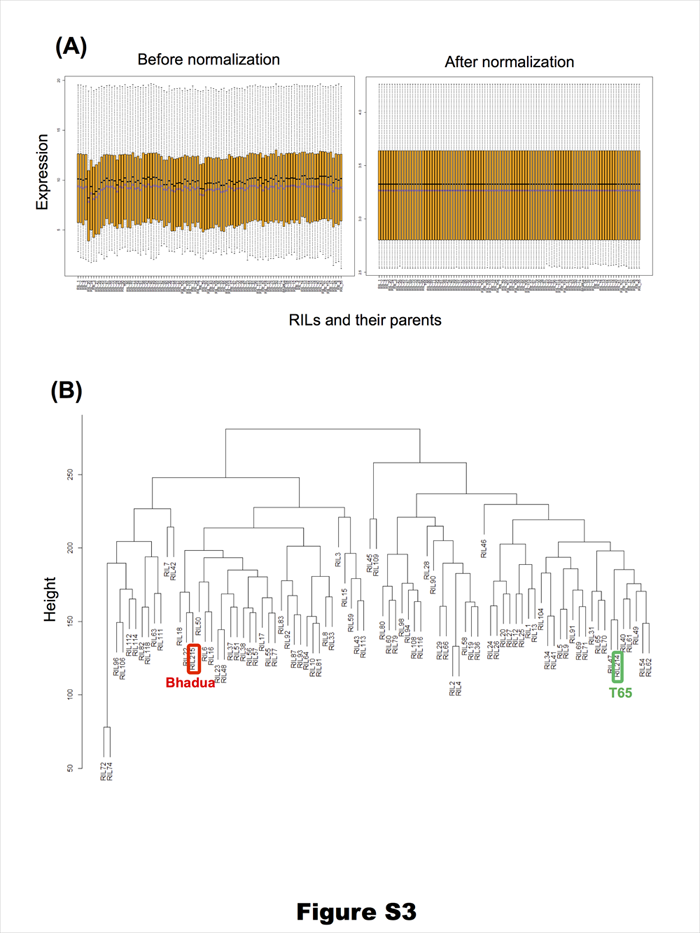

Supplement: FIGURE S3 — Overview of genome-wide transcriptome data. (A) Results of normalization of the microarray data for the RILs and their parents. (B) Dendrogram of the RILs and their parents based on microarray data. Euclidean distances and the complete linkage method were used for hierarchical clustering. [file Image_3.TIFF]

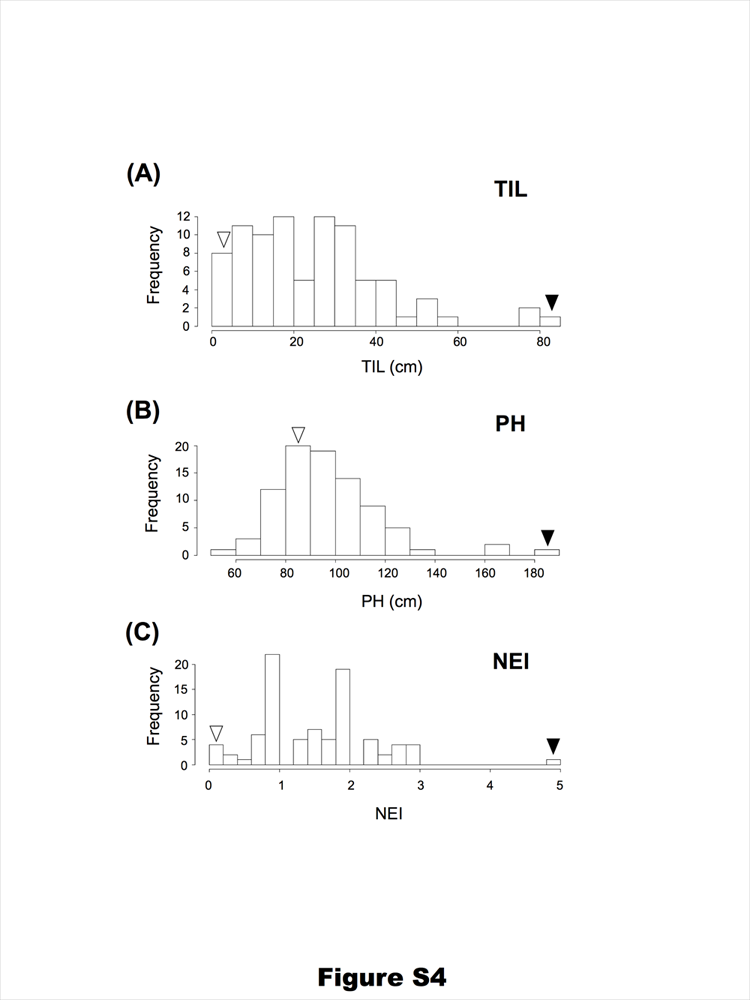

Supplement: FIGURE S4 — Statistical distribution of TIL, PH, and NEI among the RILs and their parents. (A) TIL, (B) PH, and (C) NEI are shown. White and black arrowheads represent the phenotypic values for T65 and Bhadua rice, respectively. Abbreviations: TIL, total internode length; PH, plant height; NEI, number of elongated internodes. [file Image_4.TIFF]

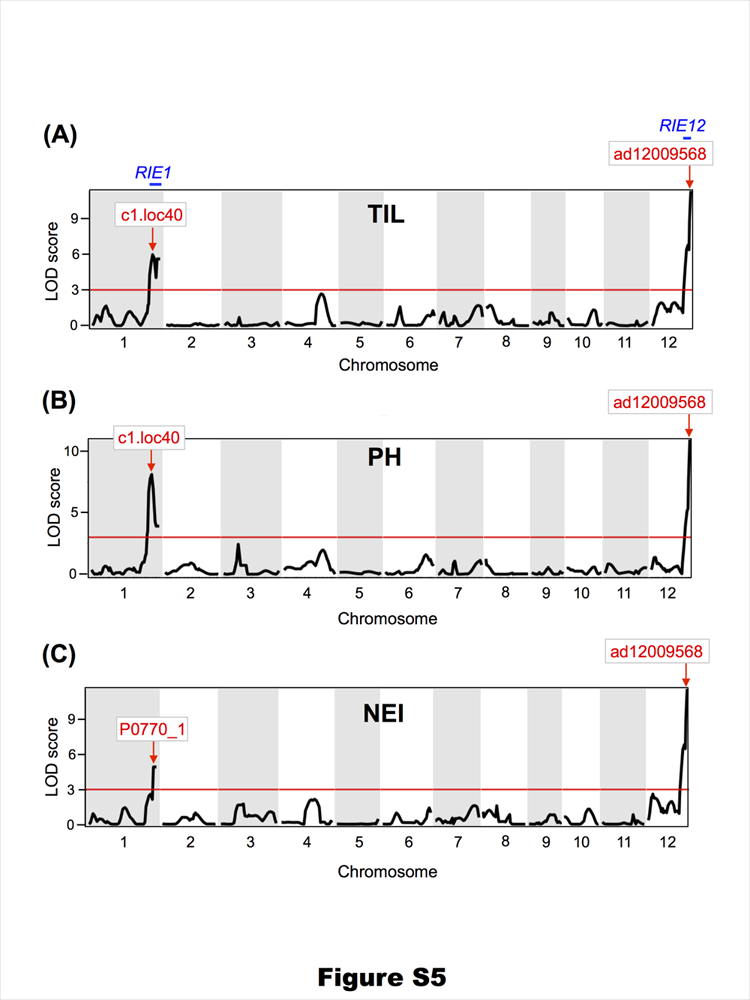

Supplement: FIGURE S5 — Overview of phQTL data of the RILs. LOD statistics of phQTLs for TIL (A), PH (B), and NEI (C). Arrows point to the pseudo-marker or marker positions. The candidate regions of qRIE1 and qRIE12 (Kawano et al., 2008) are shown with blue lines. Abbreviations: TIL, total internode length; PH, plant height; NEI, number of elongated internodes; and RIE, rate of internode elongation. The red horizontal line indicates LOD = 3. [file Image_5.TIFF]

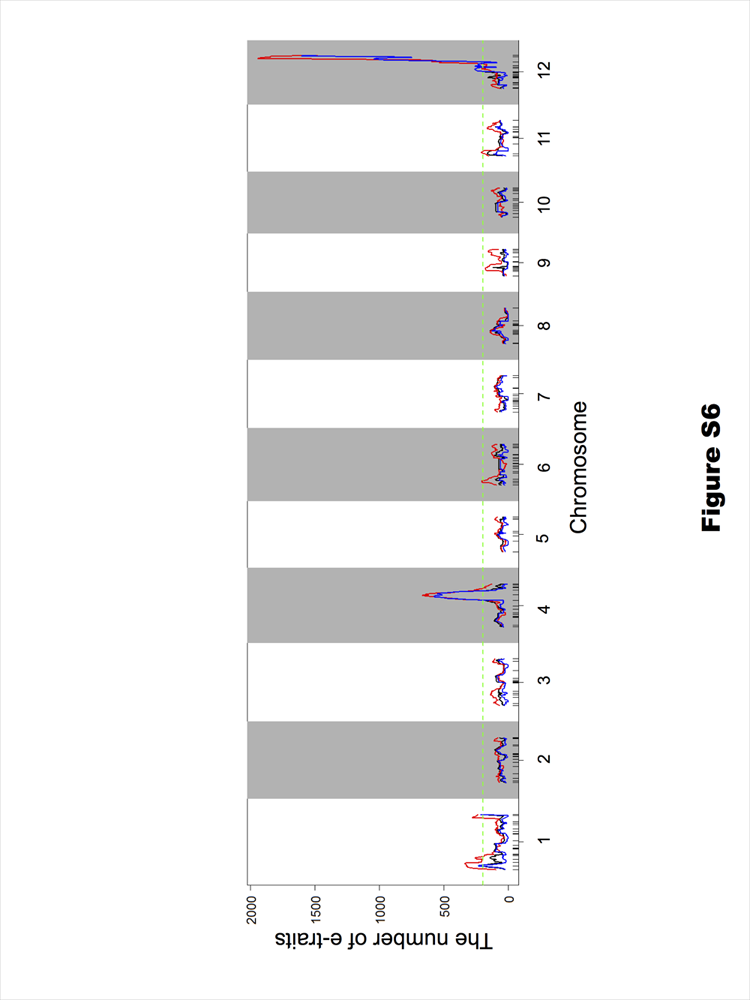

Supplement: FIGURE S6 — Overview of eQTL hotspot size significance profile. The eQTL hotspots in the 12 rice chromosomes were derived with a quantile-based permutation approach (Neto et al., 2012). Data are presented essentially as shown in Figure 4A. [file Image_6.TIFF]
